# Extended residual learning with one-shot imitation learning for robotic assembly in semi-structured environment

**DOI:** 10.3389/fnbot.2024.1355170

**Published:** 2024-04-29

**Authors:** Chuang Wang, Chupeng Su, Baozheng Sun, Gang Chen, Longhan Xie

**Affiliations:** Shien-Ming Wu School of Intelligent Engineering, South China University of Technology, Guangzhou, China

**Keywords:** object-embodiment-centric task representation, residual reinforcement learning, imitation learning, robotic assembly, semi-structured environment

## Abstract

**Introduction:**

Robotic assembly tasks require precise manipulation and coordination, often necessitating advanced learning techniques to achieve efficient and effective performance. While residual reinforcement learning with a base policy has shown promise in this domain, existing base policy approaches often rely on hand-designed full-state features and policies or extensive demonstrations, limiting their applicability in semi-structured environments.

**Methods:**

In this study, we propose an innovative Object-Embodiment-Centric Imitation and Residual Reinforcement Learning (OEC-IRRL) approach that leverages an object-embodiment-centric (OEC) task representation to integrate vision models with imitation and residual learning. By utilizing a single demonstration and minimizing interactions with the environment, our method aims to enhance learning efficiency and effectiveness. The proposed method involves three key steps: creating an object-embodiment-centric task representation, employing imitation learning for a base policy using via-point movement primitives for generalization to different settings, and utilizing residual RL for uncertainty-aware policy refinement during the assembly phase.

**Results:**

Through a series of comprehensive experiments, we investigate the impact of the OEC task representation on base and residual policy learning and demonstrate the effectiveness of the method in semi-structured environments. Our results indicate that the approach, requiring only a single demonstration and less than 1.2 h of interaction, improves success rates by 46% and reduces assembly time by 25%.

**Discussion:**

This research presents a promising avenue for robotic assembly tasks, providing a viable solution without the need for specialized expertise or custom fixtures.

## 1 Introduction

Robotics has significantly improved industrial productivity in a wide range of tasks. However, the reliance on task-specific fixtures and expert-driven programming limits the broader application of robotic assembly in settings characterized by small-batch, flexible manufacturing processes (Lee et al., 2021). These settings often present semi-structured conditions where components destined for tight-tolerance assembly are randomly oriented within a confined workspace. Such variability complicates the assembly process, demanding sophisticated manipulation skills for precise alignment and force control to ensure successful component integration.

While both model-based and learning-based methodologies have been developed to address these complexities (Suárez-Ruiz and Pham, 2016; Luo et al., 2021; Mandlekar et al., 2023), they often require prior object-specific knowledge or expensive interaction data, limiting their effectiveness and efficiency in skill acquisition. A promising way to overcome these limitations is a hybrid approach that combines the strengths of model-based and learning-based strategies, paving the way for the efficient mastery of novel tasks without necessitating robotic expertise. Recent advancements in Residual Reinforcement Learning (Residual RL) epitomize such hybrid methodologies (Johannink et al., 2019). However, challenges remain, particularly in learning full-state estimation and managing large exploration spaces for long-horizon tasks involving variable target positions and precision assembly (Carvalho et al., 2022; Wang et al., 2022).

**Figure 1:**
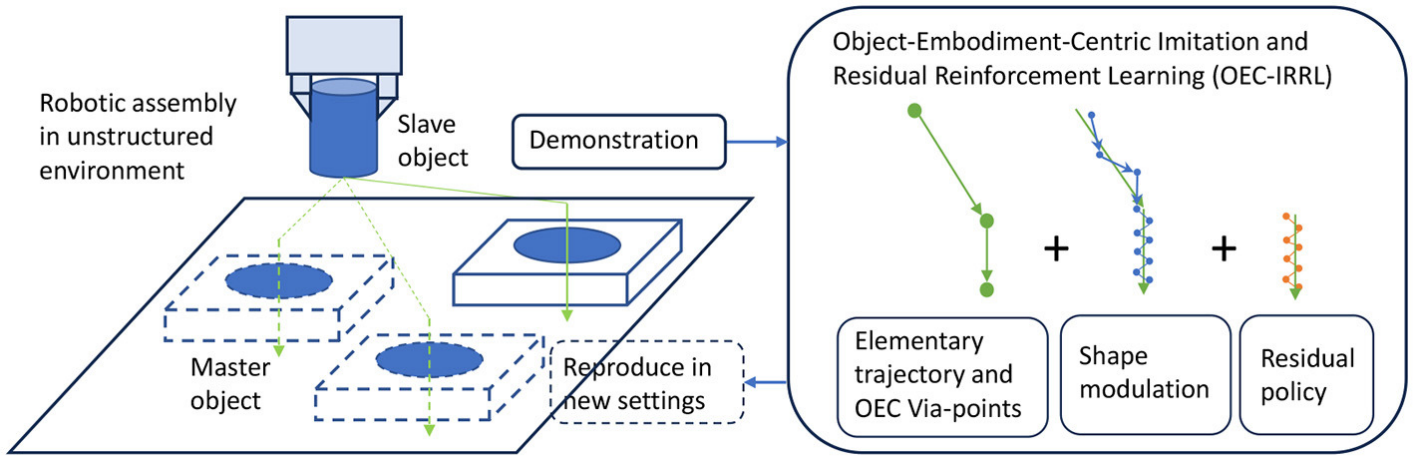
OEC-IRRL overview. We introduce an efficient and effective hybrid learning system that can perform precise assembly tasks in a semi-structured environment from a single human demonstration and less than 1.2 h of interactions.

This study aims to bridge the gap in fixture-less robotic assembly by leveraging partial knowledge of transitions to streamline robot learning (Mandlekar et al., 2023). This approach simplifies learning by segmenting it into geometry structure estimation, trajectory planning, and uncertainty handling (refer to Figure 1). It is crucial to recognize that manipulation depends on the geometric constraints of the task, the grasp pose of the slave object, and the master object's location (Li et al., 2023). Assuming the known master object's location, we can represent the motion trajectory and assembly relationship with a low-dimensional framework, facilitating skill adaptation across various poses. With the geometry structure determined, learning can concentrate on robot and task dynamics, emphasizing smooth trajectories and interaction behaviors (Shi et al., 2023). While the initial transfer phase requires only smooth trajectories, the critical assembly phase demands precise localization and intricate contact dynamics. Focused learning allows for a balanced ratio of exploitation to exploration, enhancing sample efficiency. However, integrating object-embodiment-centric partial knowledge, which simplifies the task into subtasks by encoding relevant geometric information, presents challenges: (1) extracting and representing this knowledge without robot experts, (2) incorporating it into imitation learning for efficient adaptation, and (3) balancing sub-policies for effective residual learning.

This study introduces the Object-Embodiment-Centric (OEC) task representation in an Imitation Learning (IL) and Residual RL framework, **OEC-IRRL**, which is designed for contact-rich tasks at variable locations. This framework eliminates the need for specific fixtures and extensive expert programming and enhances sample efficiency by seamlessly integrating IL and RL with partial knowledge. Our contributions are as follows: (1) Innovative Extraction of Temporal and Spatial Task Information: **OEC-IRRL** employs a via-point-based task representation to outline temporal and spatial segments of the task, enabling the learning of adaptive operations from a single demonstration and acceptable interactions. We extract via-points from the demonstrated trajectory based on velocity, dividing the task into transfer and assembly phases. The OEC task representation includes the start via-point in the robot base frame, as well as the middle and end via-points in the master object frame, offering essential geometry information without extensive robot calibration or task-specific knowledge. This is particularly useful in dynamic environments where the master object's pose is estimated by a vision model. (2) Guided Hybrid IL and Residual RL for Enhanced Learning Efficiency: This novel approach uses the OEC representation to guide efficient learning through VMP and limits the exploration range of residual RL. Improved VMPs learn the motion trajectory from demonstrations and via-points, where the basic trajectory encodes via-point geometry and shape modulation dictates the trajectory distribution for smooth exploration. This strategy allows for adaptation to various settings while keeping the trajectory profile consistent during assembly. Moreover, residual RL is selectively applied in the assembly phase for precise localization and contact dynamics, minimizing exploration space for efficient learning and reusing policies across locations under the base policy's guidance. The exploration behavior learned from human demonstrations notably increases success rates. (3) Experiment Validation of OEC Task Representation and Framework: Through extensive testing, we have shown that OEC task representations can be effectively derived from a single demonstration, greatly enhancing the sample efficiency of VMP-based IL and multimodal residual RL in extended tasks. Our experiments confirm the learned strategies' applicability to various fixtureless assembly tasks across different locations, significantly advancing robotic assembly.

## 2 Related work

Deep reinforcement learning (DRL) techniques have become increasingly popular for contact-rich activities due to their potential to provide an alternative to the complicated and computationally expensive process of modeling intricate environments. Despite its potential, the application of DRL to complex manipulation tasks has been hampered by issues related to sample efficiency and safety. To mitigate these challenges, previous task-specific knowledge has been exploited, including bootstrapping from demonstrations through a specific teleoperation system in the study by Nair et al. (2018), utilizing high-performance simulators for sim2real in the study by Amaya and Von Arnim (2023), and exploiting knowledge of similar tasks by pre-training on the task family in the study by Hao et al. (2022). Although these strategies have shown the potential to improve sample efficiency and ensure safer DRL applications, extracting and using prior knowledge requires a lot of engineering effort. Therefore, this section discusses methods that extend RL to perform accurate assembly tasks in semi-structured environments via a base policy, which is accessible in manufacturing.

### 2.1 Model-based base policy

Residual RL was originally proposed to integrate conventional controllers with DRL to solve complex manipulation tasks. RL is utilized to handle the unknown aspects of the task, while a hand-designed controller manages the known elements in the study by Silver et al. (2018); Johannink et al. (2019). This integration simplifies controller design and improves sample efficiency. Different controllers and integration techniques have been examined in the current literature. Schoettler et al. (2020) applied Residual RL in real-world industrial tasks using a hand-designed P-controller as the base policy. In contrast, Beltran-Hernandez et al. (2020) concentrated on learning force control for position-controlled robots using a state-based controller gain policy. Additionally, Ranjbar et al. (2021) proposed a hybrid residual RL approach and aimed at modifying both the feedback signals and the output via the RL policy to prevent the low-level controller's internal feedback signals from restricting the RL agent's capacity to optimize its policy, thus hindering learning.

Visual servoing and motion planning have played a crucial role in guiding DRL methods in unstructured environments. Shi et al. (2021a) have introduced a visual RL method that unites a fixed visual-based policy and a parametric contact-based policy, guaranteeing a high success rate in the task and the capacity to adapt to environmental changes. Meanwhile, Lee et al. (2020) quantify uncertainty in pose estimation to determine a binary switching strategy using model-based or RL policies. Additionally, Yamada et al. (2023) implemented an object-centric generative model to identify goals for motion planning and a skill transition network to facilitate the movement of the end-effector from its terminal state in motion planning to viable starting states of a sample-efficient RL policy. However, these methods require the model of the object, in particular, the manual specification of a goal state in the robot's frame and control policy design (Yamada et al., 2023). Additionally, they face difficulties in providing comprehensive guidance in both free space and contact-rich regions due to the limited motion planning in tasks that require environmental interaction and the scarcity of visual servoing in addressing geometric constraints.

### 2.2 Imitation learning-based base policy

Leveraging prior knowledge in the form of demonstrations can extend the application of residual RL to scenarios where accurate state estimation and first-principle physical modeling are not feasible (Zhou et al., 2019; Wang et al., 2023). Mathematical model-based movement primitive (MP) with compact representation is a promising method for learning controllers that can solve the non-linear trajectories from a few human demonstrations. For instance, Ma et al. (2020) recently presented a two-phase policy learning process that employs a Gaussian mixture model (GMM) as a base policy to accelerate RL. Davchev et al. (2022) introduced a framework for employing full pose residual learning directly in task space for Dynamic Movement Primitives (DMP) and demonstrated that residual RL outperforms RL-based learning of DMP parameters. Carvalho et al. (2022) investigated the use of variability in demonstration of Probabilistic Movement Primitives (ProMP) as a decision factor to diminish the exploration space for residual RL. They compared this method with a distance-based strategy. Neural networks are also used well for imitation learning methods in residual RL. Wang et al. (2022) have developed a hierarchical architecture for offline trajectory learning policies, which are complemented by a reinforcement learning-based force control scheme for optimal force control policies.

Visual imitation learning is essential to enable residual RL of difficult-to-specify actions under diverse environmental conditions. Alakuijala et al. (2021) suggest learning task-specific state features and control strategies from the robot's visual and proprioceptive inputs using behavioral cloning (BC) and convolutional neural network (CNN) on demonstrated trajectories for residual RL. The resulting policy can be trained solely using data, which is demonstrated for the base controller and with rollouts in the environment for the residual policy. However, achieving generalization through adaptable control strategies and state estimation from high-dimensional vision information requires a significant number of demonstrations. Additionally, to prevent unnecessary exploration in free space regions, the activation decision of the residual policy needs to be closer to the assembling phase and rely on trajectory distributions from numerous demonstrations or task-specific knowledge for geometric constraints.

In response to these challenges, this study proposes a novel OEC task representation within imitation learning (IL) and residual RL frameworks, which are tailored to enable the learning of adaptive operations from minimal demonstrations and interactions. This approach builds upon these foundations of the prior vision model from the model-based methods (Lee et al., 2020; Shi et al., 2021a; Yamada et al., 2023) and the mathematical model from the imitation learning-based methods (Carvalho et al., 2022; Davchev et al., 2022). Our approach distinguishes itself by: (1) streamlining robot programming through extracting via-points from demonstrated end-effector trajectories for task representation, thereby simplifying the reconfiguration costs and improving adaptability. (2) Integrating IL and Residual RL to effectively manage both free space and contact-rich regions, overcoming the limitations of previous approaches in terms of learning efficiency and effectiveness. In contrast to the study by Mandlekar et al. (2023), Zang et al. (2023) using the base policy for data augmentation, this study uses residual RL for further optimization on the base policy.

## 3 Problem statement

In this study, we formalize contact-rich assembly tasks in a semi-structured environment as a Markov Decision Process (MDP), *M* = (*S, A, P, r*, γ, *H*). For a system with the transition function *P* and reward function *r*, we want to determine a policy π, which is a probability distribution over actions *a*∈*A* conditioned on a given state *s*∈*S*, to maximize the expected return $\overset{H}{\underset{t=0}{\sum }}\gamma^{t}r$ in the rollout with a horizon of *H*.

The assumption employed in this study can be stated as having partial knowledge of the transition function *P* (Lee et al., 2020), including a two-stage operation process and a coarse estimation of the environmental state. The policy is typically formulated from a combination of sub-policies, which may depend on time and state as Equation (1) (Johannink et al., 2019; Davchev et al., 2022):


(1) $$\begin{array}{l} \pi \left(a|s,t\right)=\alpha \left(s,t\right)\pi_{b}\left(s,t\right)⊕\beta \left(s,t\right)\pi_{\theta }\left(a|s,t\right) \end{array}$$

where π_*b*_ is a base policy (offline learning or model-based), π_θ_ is an online learning-based policy, and α and β are the adaptation parameters. The operation ⊕ depends on the integration method.

By leveraging a precomputed offline continuous base policy, π_*b*_, the task complexity for π_θ_ is significantly reduced (Carvalho et al., 2022). Thereafter, the residual policy is tasked with learning how to deviate from the base policy to overcome model inaccuracies and potential environmental changes during execution. The final policy can mitigate system uncertainties and ensure contact safety through adaptation parameters. To optimize the objective derived from the sampled trajectories, a policy gradient method is implemented to update the π_θ_.

A key question in this context is how to obtain the π_*b*_ and adaptation parameters to guide π_θ_. The proposed methodology entails directly acquiring them in task space from a demonstrated trajectory and a prior vision model, as described in the following section.

## 4 Method

**Figure 2:**
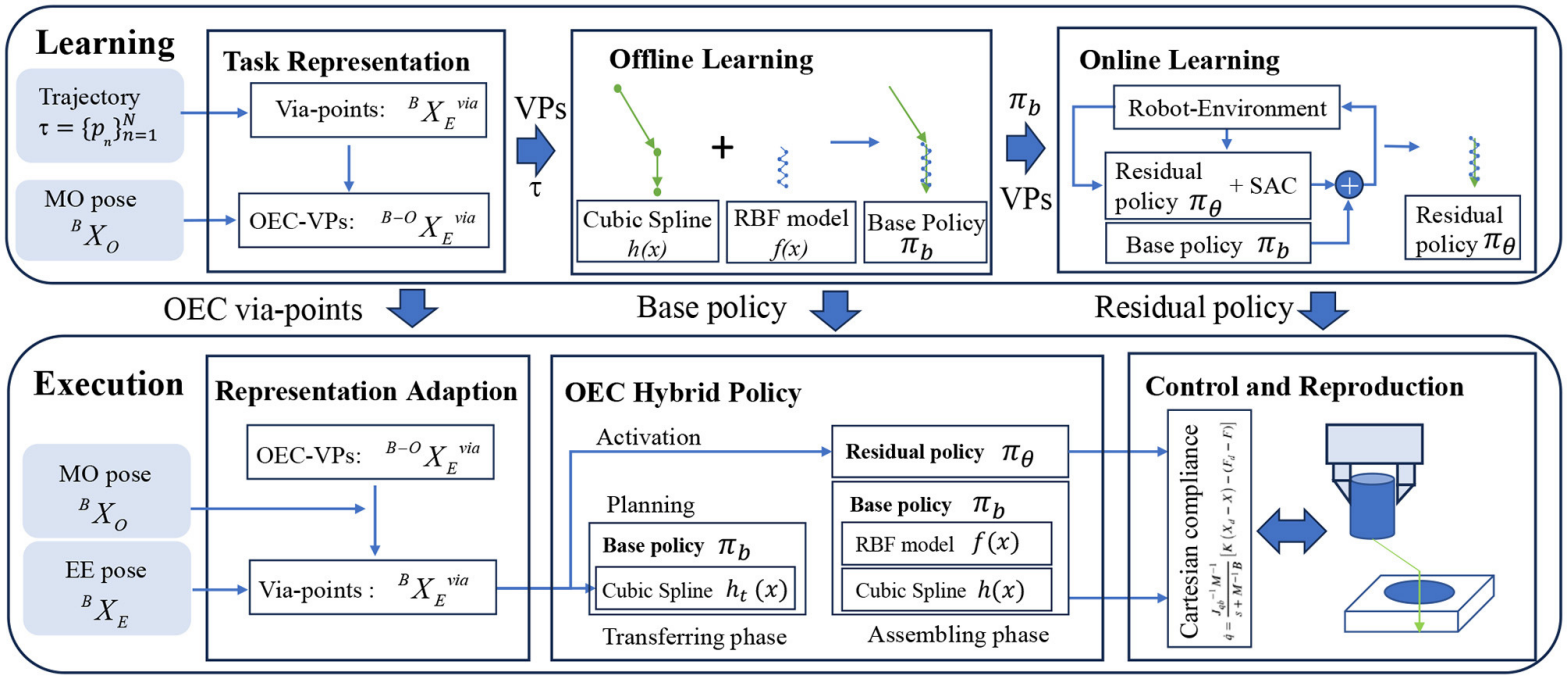
System overview. The first step is to extract structured information from the demonstration using the OEC task representation. Then, the OEC task representation is used to plan the elementary trajectory in the offline IL, with the dynamic behavior in the demonstration encoded by shape modulation. Finally, the residual policy is selectively activated by the OEC task representation to concentrate on the uncertainty during assembly. To adapt to different poses, the base trajectory is revised using an adaptive OEC task representation which directs invariant dynamic behavior and handles uncertainty, enabling the reproduction of assembly skills.

This study introduces an OEC-IRRL framework for precise assembly tasks without specific fixtures (see Figure 2 for an overview). It encompasses a coarse operation for long-horizon exploration and a fine operation for uncertainty compensation. The OEC-IRRL method begins by pre-processing the recorded data from a single demonstration trajectory of the end-effector $\tau ={\left[X_{n}\right]}_{n=1}^{N}$ and the master object pose ${\;}^{B}X_{O}$ obtained from an eye-to-hand camera. This pre-processing step involves the generation of the OEC task representation, which enables efficient learning policies that adapt to new settings. Via-points (VPs) are extracted from the trajectory based on the velocity and then converted into OEC-VPs representing task robot-related temporal and spatial information (Section 4.1). Subsequently, a base policy (π_*b*_) based on piece-wise VMP is fitted using the VPs and trajectory to facilitate coarse movements, including transferring and assembling (Section 4.2). Leveraging π_*b*_ and VPs, a multimodal residual policy (π_θ_) is learned through RL to enable precise localization and variable force control in contact-rich tasks (Section 4.3). Following the learning process, the obtained sub-policies (OEC-VPs, π_*b*_, and π_θ_) and the current state (including master object pose ${\;}^{B}X_{O}$ and end-effector pose ${\;}^{B}X_{E}$) are utilized for skill execution. New VPs are obtained from OEC-VPs by representation adaptation. The π_*b*_, after shape modulation by VPs, guides the robot in both free space and contact-rich regions. The π_θ_ is selectively activated by the VPs in contact-rich regions, working in conjunction with the parallel position/force controller to effectively reproduce the demonstrated skill (Section 4.4).

### 4.1 Task representation

Demonstration-based programming has been proposed to handle variations in geometry with less engineering effort in robot calibration and task-specific reconfiguration (Shi et al., 2021b). The goal of this section is to extract and define an OEC task representation with a single demonstration and a prior vision model, which provides the task and robot-related information for efficient learning in long-horizon tasks and adaptability to variable positions in a semi-structured environment.

This study equips an eye-to-hand camera to provide a global view of the workspace, capturing a 2D image denoted *I*_*eth*_. The relative pose of the master object in the robot's base frame ${\;}^{B}X_{O}$ can be obtained from extrinsic and intrinsic camera parameters by hand-eye calibration and YOLO-based detectors fine-tuned to the domain. The YOLO algorithm is widely used to detect objects in the image or video streams (Mou et al., 2022). For each object in the image *I*_*eth*_, the algorithm makes multiple predictions of bounding boxes that contain information concerning the object's position (*x, y*), size (*w, h*), confidence *c*_*con*_, and category *c*_*cate*_, as shown in Equation (2). The algorithm selects the most effective predicted bounding box for the object based on a predefined confidence level.


(2) $$\begin{array}{l} \left[c_{cate},x,y,w,h,c_{con}\right]=YOLO\left(I_{eth}\right) \end{array}$$

A perception system based on object detection generates a bounding box around the master object to obtain the location (*x*_0_, *y*_0_), and two additional bounding boxes are generated around the predefined feature structures to obtain the locations (*x*_1_, *y*_1_) and (*x*_2_, *y*_2_). Using the eye-to-hand transformation ${\;}^{B}T_{C}$, the estimated points $\left(x_{i}^{\prime },y_{i}^{\prime }\right)$ are converted to the robot frame, as shown in Equation (3). The partial pose information of the master object, including its orientation in *Rx* and *Ry* and translation in *z* dimensions *z*_*con*_, is taken into account to determine the pose ${\;}^{B}X_{O}$, as shown in Equation (4). The calculated position is accompanied by an error *E*_*r*_.


(3) $$\begin{array}{l} \left(x_{i}^{\prime },y_{i}^{\prime }\right){=}^{B}T_{C}\left(x_{i},y_{i}\right),i=0,1,2 \end{array}$$

(4) $${\;}^{B}X_{O}=[x_{0^{\prime }},y_{0^{\prime }},z_{con},0,0,\arctan(\frac{y_{2^{\prime }}-y_{1^{\prime }}}{x_{2^{\prime }}-x_{1^{\prime }}})]+E_{r}$$

**Figure 3:**
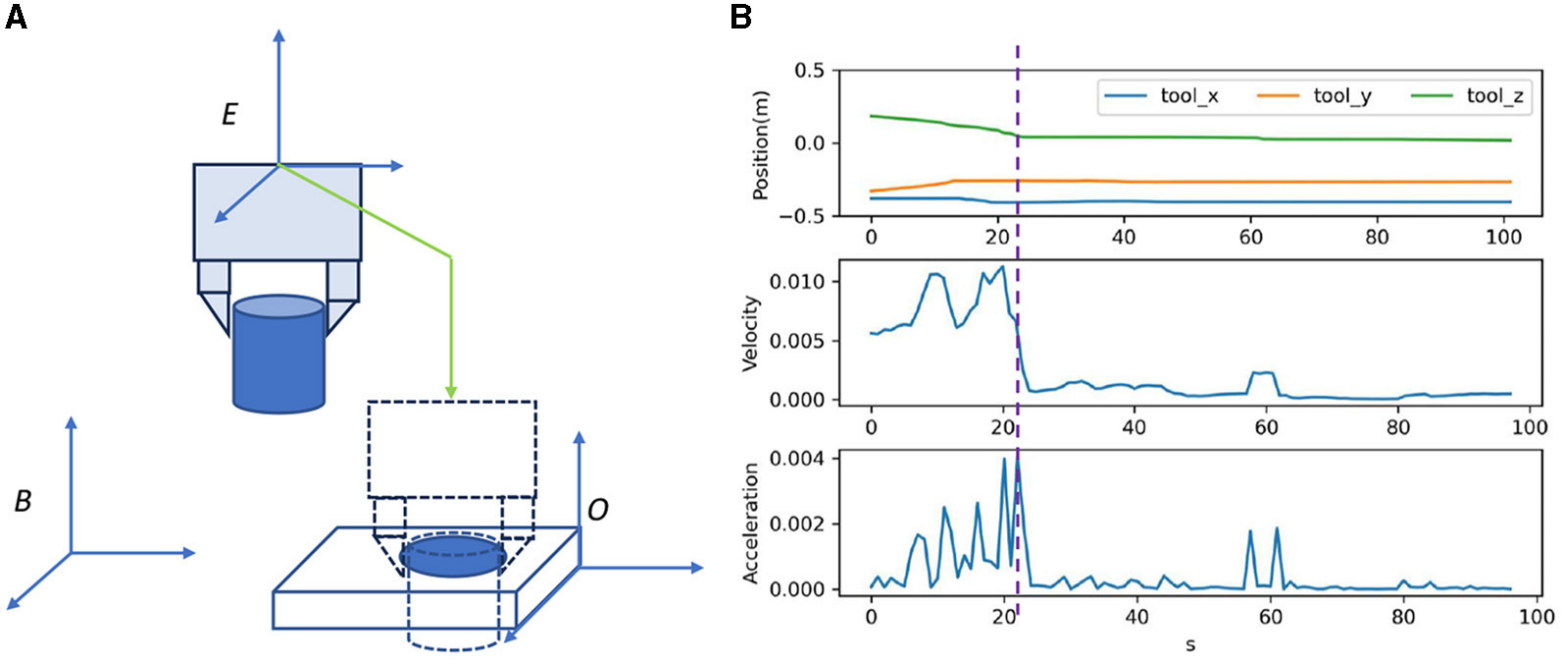
OEC task representation. The trajectory is demonstrated as shown in **(A)** and analyzed as shown in **(B)** for OEC task representation.

The demonstration is performed by a tele-operation system, which first moves the slave object to the master object and then assembles them, as shown in Figure 3. In the demonstration, ${\;}^{B}X_{O}^{0}$ observed at the start and the trajectory of the end-effect ${\;}^{B}X_{E}^{i}$ at each step *T*^*i*^ is recorded $\tau ={\left[\left(T^{i}{,}^{B}X_{E}^{i}{,}^{B}X_{O}^{0}\right)\right]}_{n=1}^{N}$.

To reduce the exploration horizon, this study analyzes the assembly process and extracts the bottleneck pose for task segments. Although various techniques, such as dynamic programming algorithm (Shi et al., 2023) or stochastic-based method (Lee et al., 2019), have been used for automatic waypoint extraction, this study uses a simpler method of velocity-based motion mode switch detection (VMMSD), which is motivated by the instinctive switching between fast arrival and safe fine-grained operation behavior modes, as shown in Figure 3. First, we define *P* as the 3-d translation of ${\;}^{B}X_{E}$ for the bottleneck position estimation. Second, we estimate the nominal velocity $v={\left[\left(T^{i},v^{i}\right)\right]}_{n=1}^{N}$ and smooth it using a moving average, as shown in Equation (5). The pose with the highest velocity change serves as the bottleneck poses ${\;}^{B}X_{E}^{m}$, as shown in Equation (6), which divides the skill into transferring in the free space and assembling in the contact-rich region.


(5) $$\begin{array}{l} \hat{v}=\text{convolve}\left(v,w\right),v^{i}=\frac{P^{i}-P^{i-1}}{T^{i}-T^{i-1}} \end{array}$$

(6) $$\begin{array}{l} m=\text{argmax}\left(a\right),a^{i}=\frac{{\hat{v}}^{i}-{\hat{v}}^{i-1}}{T^{i}-T^{i-1}} \end{array}$$

where *w* is the moving average window, *a* is the nominal acceleration, and *m* is the bottleneck position index.

For temporal and spatial adaptation, we have established an OEC task representation for learning. We first define the via-points ${\;}^{B}X_{E}^{via}$ to represent structured information. Together with the extracted bottleneck pose ${\;}^{B}X_{E}^{m}$, the start pose ${\;}^{B}X_{E}^{s}$ and the goal pose ${\;}^{B}X_{E}^{g}$ are specified as the first and last poses of the trajectory, as shown in Equation (7). A canonical variable *t* serves as a virtual timer, linearly increasing from 0 to 1 in this study. We then transform the bottleneck and goal pose in via-points from the robot base frame into the task frame using the master object pose estimated by the object detection model, as shown in Equation (8). This allows the task robot-related information to be scaled to scenes with different robot and master object poses.


(7) $${\;}^{B}X_{E}^{\text{via}}=[(0{,}^{B}X_{E}^{s}),(t_{m}{,}^{B}X_{E}^{m}),(1{,}^{B}X_{E}^{g})],t_{m}=\frac{m}{H}$$

(8) $${\;}^{B-O}X_{E}^{\text{via}}={[}^{B}X_{E}^{s}{,}^{O}X_{E}^{m}{,}^{O}X_{E}^{g}]={[}^{B}X_{E}^{s},{{(}^{B}X_{O})}^{-1}{(}^{B}X_{E}^{m}{,}^{B}X_{E}^{g})]$$

### 4.2 Offline learning

In semi-structured environments, a concise trajectory representation is required to encode geometry constraints and motion dynamics related to the task and robot while being adaptable to various target positions. Therefore, this section presents OEC piece-wise VMP and demonstrates the importance of the bottleneck pose in via-points.

Motion primitives are commonly employed to model movements in few-shot imitation learning. In this study, VMP is used due to the enhanced capability of via-point modulation compared with DMP and ProMP (Zhou et al., 2019). The VMP method combines a linear elementary trajectory *h*(*t*) with a non-linear shape modulation *f*(*t*), as shown in Equation (9).


(9) $$\begin{array}{l} y\left(t\right)=h\left(t\right)+f\left(t\right) \end{array}$$

where t is the canonical variable increasing linearly from 0 to 1, and y is the generated current pose.

It is assumed that the elementary trajectory *h*(*t*) serves as the fundamental framework alongside the extracted via-points. The cubic spline is a commonly used interpolation technique which ensures that the position and velocity curves remain continuous, which is equivalent to the goal-directed damped spring system of DMP. The elementary trajectory can be obtained as Equation (10).


(10) $$\begin{array}{l} h\left(t\right)=\overset{3}{\underset{k=0}{\sum }}a_{k}t^{k} \end{array}$$

The parameters *a*_*k*_ results from the four constraints, as shown in Equation (11).


(11) $$h(t_{0})=y_{0},\dot{h}(t_{0})={\dot{y}}_{0},h(t_{1})=y_{1},\dot{h}(t_{1})={\dot{y}}_{1}$$

where (*t*_0_, *y*_0_) and (*t*_1_, *y*_1_) are two adjacent via-points.

The shape modulation term *f*(*t*) encodes the dynamic behavior of the demonstrated trajectory. It is explained as a regression model consisting of *N*_*k*_ squared exponential kernels, as shown in Equation (12).


(12) $$\begin{array}{l} f\left(t\right)=\text{Ψ}{\left(t\right)}^{T}\omega ,\psi_{i}=\text{exp}\left(-h_{i}{\left(t-c_{i}\right)}^{2}\right),i\in \left[1,N_{k}\right] \end{array}$$

where *h*_*i*_ and *c*_*i*_ are predefined constants. Similar to ProMP, VMP assumes that the weight parameter ω~*N*(μ, σ) follows a Gaussian distribution. The parameter ω can be learned via maximum likelihood estimation (MLE) from the trajectory between *t*_0_ and *t*_1_.

To handle intermediate via-point, we divide the trajectory into segments to create piece-wise VMP, as shown in Equation (13). In particular, we only use *h*(*t*) during the transfer phase, which leads the robot through free space and disregards the suboptimal curved trajectory.


(13) $$y(t)=\{\begin{array}{ll} h_{t}(t),t_{0}=0,t_{1}=t_{m} & t\leq t_{m}\\ h_{a}(t)+f_{a}(t),t_{0}=t_{m},t_{1}=1 & t>t_{m} \end{array}$$

This study implements via-point modulation to adapt to different positions by manipulating the elementary trajectory, *h*(*t*), using the OEC task representation.

**Figure 4:**
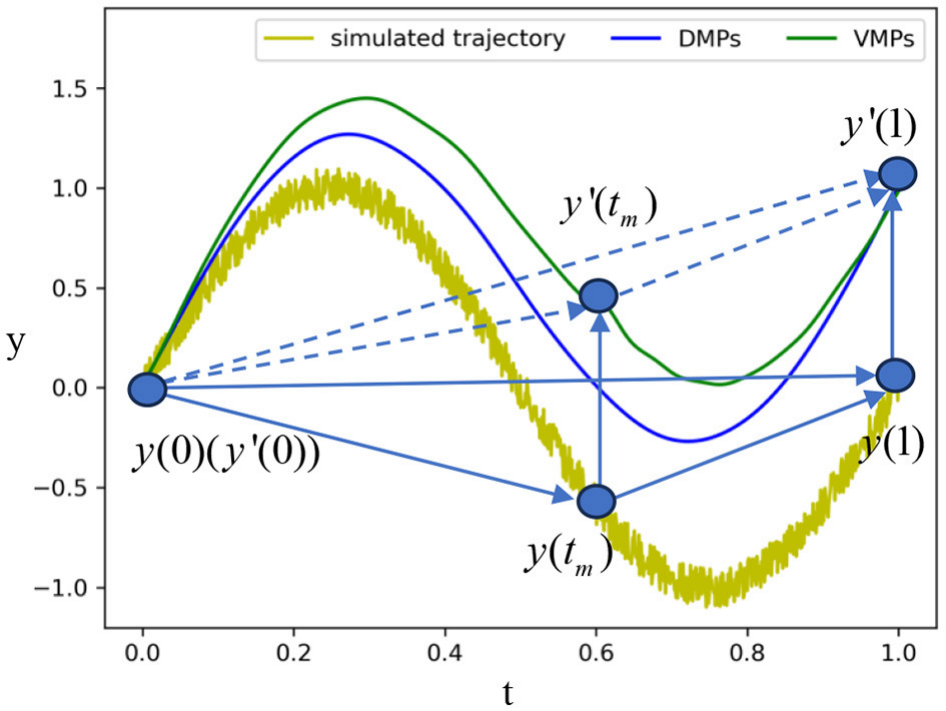
Comparison of VMP and DMP in scaling to a new goal position. The yellow line is a simulated demonstration using a sine wave trajectory and Gaussian noise. The blue curve is the scaled trajectory using DMP without a middle via-point. The green curve is the scaled trajectory with the middle via-point.

To investigate the effect of via-points on the reproduction results (Wang et al., 2022), we introduce a translation to the goal pose in the VMP formulation of a sine wave, as depicted in Figure 4. The yellow line represents a sine wave trajectory with Gaussian noise. We spatially scale the sine wave to match a new goal *y*′(1) using one-dimensional VMP. The blue curve represents the scaled trajectory using vanilla VMP (DMP), i.e., no mid-point is considered. With such a baseline, we then add a bottleneck pose to the VMP formulation and show the scaled trajectory as the green curve. The results indicate that the bottleneck pose can maintain the invariant trajectory of assembling in scaling. As the relative position of the start and goal points varies, the trajectory profile of the blue curve is changed, while the middle via-point maintains the unchanged part between itself and the goal.

### 4.3 Online learning

The residual policy is learned from interaction under exploration guidance to compensate for uncertainties in position and contact dynamics. Together with the OEC task representation, the learned VMP guides the RL in two ways, exploration range and distribution in the contact-rich region. Different from Jin et al. (2023), this study jointly trains vision-force fusion and policy by an error curriculum learning for robust residual policy in the insertion task.

Compliance enables a trade-off between tracking accuracy and safety requirements, especially active compliance is particularly useful in making system dynamics more easily adjustable (Schumacher et al., 2019). Based on the mass-spring-damper model, a basic parallel position/force controller is utilized as the low-level controller to integrate the two components of the assembly policy, thereby generating a velocity command. The absence of integral and differential terms ensures that both the force and trajectory strategies have equal importance, rather than excessively prioritizing positional accuracy. The robot exhibits compliance and directly learns the operation skills in the task space. The control law for joint velocities $\dot{q}$ can be given as Equation (14).


(14) $$\begin{array}{l} \dot{q}=\frac{J^{-1}M^{-1}}{s+M^{-1}B}\left[K\left(X_{d}-X\right)-\left(F_{d}-F\right)\right] \end{array}$$

where *J* is the Jacobian matrix, which maps joint velocities to end-effector velocities. *M*, *B*, and *K* are the virtual mass, damping, and stiffness matrices. *F* and *F*_*d*_ are the measured interaction force and desired force. *X* and *X*_*d*_ are the current pose and desired reference pose.

This study formulated the combination of base and residual sub-policies based on the compliance controller in task space as Equation (15).


(15) $$\begin{array}{l} \pi \left(a|s,t\right)=K\left(t\right)\left(\pi_{b}\left(t\right)-X_{t}\right)+\beta \left(t\right)\left(\pi_{\theta }\left(a|s,t\right)-F_{t}\right) \end{array}$$

where stiffness *K*(*t*) and selective activation β(*t*) work as adaption parameters.

To enable effective decision-making in residual policy, multimodal information is fused to uniquely identify the states of physical contact with the environment, and stochastic policy representation is used to balance exploration and exploitation. The multimodal policy consists of two elements. The two-layer Long-Short-Term Memory (LSTM) network with 64 hidden nodes is used to extract 6-dimensional latent features from time-series contact force [*F*_*t*−*n*_, ..., *F*_*t*_] and relative pose [*Rp*_*t*−*n*_, ..., *Rp*_*t*_]. A three-layer convolutional neural network (CNN) converts the high-dimensional visual information *I*_*eih*_ into a corresponding 6-dimensional feature vector. After that, a multilayer perceptron (MLP) is employed to integrate the low-dimensional latent features and generate Gaussian distributions for action sampling. The action *a* of stochastic policy is mapped to the desired force *F*_*d*_ as the input of the position/force controller based on the estimated safe contact force range ${F_{d}}^{\max}$, which is defined as Equation (16).


(16) $$\begin{array}{l} F_{d}=diag\left(a\right)\cdot {F_{d}}^{\max} \end{array}$$

Our choice of reinforcement learning (RL) method is Soft-Actor-Critic (SAC), which is considered to be a state-of-the-art model-free approach. SAC is a deep RL algorithm of the off-policy actor-critic type, based on the maximum entropy reinforcement learning framework. It aims to maximize the expected reward while optimizing maximum entropy. The reward is defined by the goal pose ${\;}^{B}X_{E}^{g}$, and distance and force punishment reward shaping is employed to balance between efficient and gentle behavior.

The trajectory τ can be divided into two phases: transfer in free space and assembly in contact-rich regions using via-points from the demonstration. This approach ensures low tracking error in free space, aided by a large stiffness *K*_*h*_, and a low gain *K*_*l*_ is required for safety during assembly with limited tolerance. Nevertheless, uncertainties and low gains prevent the controller from perfectly following the desired trajectory, resulting in the inability to complete the task. This study uses learning from interaction with exploration guidance to compensate for the uncertainties within the contact-rich region. Therefore, the stiffness switch and learning process are activated at the bottleneck pose *t*_*m*_ for efficient and safe learning, as shown in Equation (17).


(17) $$K(t),\beta (t)=\{\begin{array}{ll} K_{h},0 & t\leq t_{m}\\ K_{l},1 & t>t_{m} \end{array}$$

The error curriculum is used to allow the agent to first concentrate on managing accurate localization and contact dynamics and then enhancing robustness to random position error in unfixed insertion tasks. With an initial *Er*_0_, the error increases δ*r* as the success rate *sr* reaches *a* and decreases as it reaches *b*, as shown in Equation (18). The error *e* is introduced to the base policy as Gaussian noise, as shown in Equation (19).


(18) $$\begin{array}{l} Er_{i+1}=Er_{i}+\delta r_{\left(sr>a\right)}-\delta r_{\left(sr\leq b\right)} \end{array}$$

(19) $$\begin{array}{l} \pi_{b}\left(t\right)=\pi_{b}\left(t\right)+e,e∽Gaussian\left(0,Er\right) \end{array}$$

**Figure 5:**
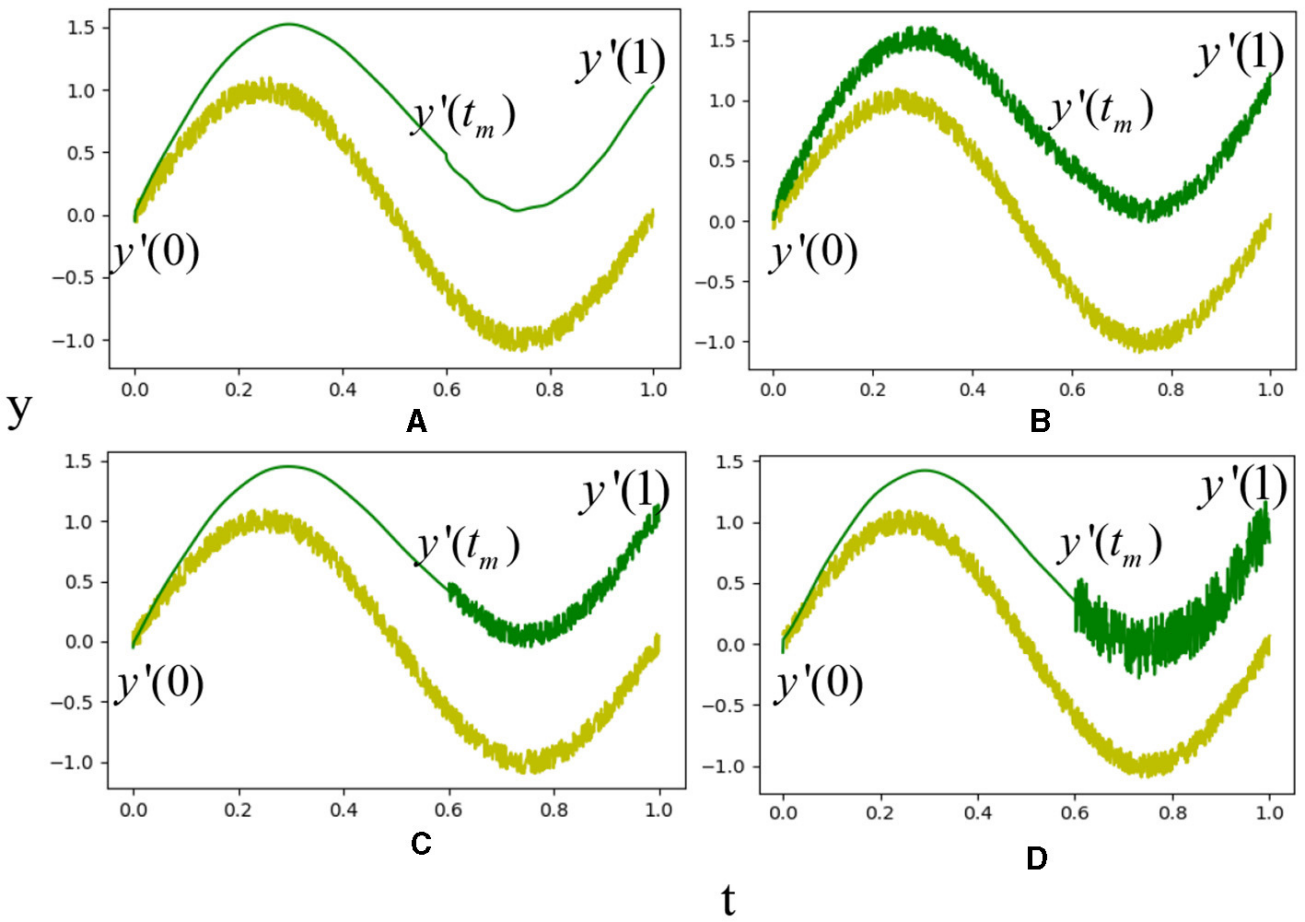
Selective activation and error curriculum for residual learning. The yellow and green curves in **(A)** are the simulated demonstration and scaled trajectory. The profile around the green curves in the figure shows the search space of residual policy with full activation in **(B)**, selective activation in **(C)**, and error curriculum in **(D)**.

To investigate the effect of residual policy and adaptation parameters on the motion results, we introduced a random residual policy on the scaled trajectory with different *K*(*t*) and β(*t*), as shown in Figure 5. The green curve in the first subfigure indicates the scaled trajectory. We activate the residual policy at the start point in Figure 5B and at the middle point in Figure 5C. The residual policy is activated using weekly trajectory guidance, as shown in Figure 5D. The figure displays the exploration space of the residual policy, with the profile surrounding the green curves. The results demonstrate that the VMP with a middle via-point provides more effective guidance, taking into account the geometric constraint. The exploration space can be further narrowed through selective activation and an error curriculum, utilizing uncertainty-aware exploration.

### 4.4 Skill execution

Using the OEC-IRRL framework and learned sub-policies, task execution can be completed at variable target locations within the workspace. Execution follows three steps: representation adaptation, elementary trajectory replanning for transferring, and hybrid policy activation for assembling.

With the current pose of the master object ${\;}^{B}X_{O}$ and the end-effector pose ${\;}^{B}X_{E}$ in the robot base frame, the OEC-VPs ${\;}^{B-O}X_{E}^{via}$ can be transformed into via-points in the robot base frame ${\;}^{B}X_{E}^{via}$. After that, the via-points ${\;}^{B}X_{E}^{via}$ are used to replan the elementary trajectory of the VMP in the current scene. The reproduced desired trajectory guides the robot's end-effector to the location of the master object by the compliance controller with high stiffness in free space. After *t*_*m*_, the stiffness is switched, and the residual RL policy is activated to handle the uncertainties caused by pose estimation, demonstration, and execution.

## 5 Experiments

These experiments evaluate the effectiveness of the OEC task representation in scaling the demonstrated trajectory to a variable goal pose using VMP and in appropriately activating the residual policy for efficient residual learning. This section presents the experimental setup, comparison with existing work, and experiments to evaluate the proposed approach. The experiments are structured into four parts. First, the VMMSD is evaluated by detecting the bottleneck pose in demonstrations from various poses and tasks. Second, the piece-wise VMP approach is used to learn the operational trajectory and assess its robustness in scaling different positions. Third, the effect of the activation point on the learning efficiency of the residual RL is analyzed on a gear insertion task. Finally, the hybrid policy is evaluated by comparing OEC-IRRL with three other baselines in a semi-structured environment.

### 5.1 Experimental setup

**Figure 6:**
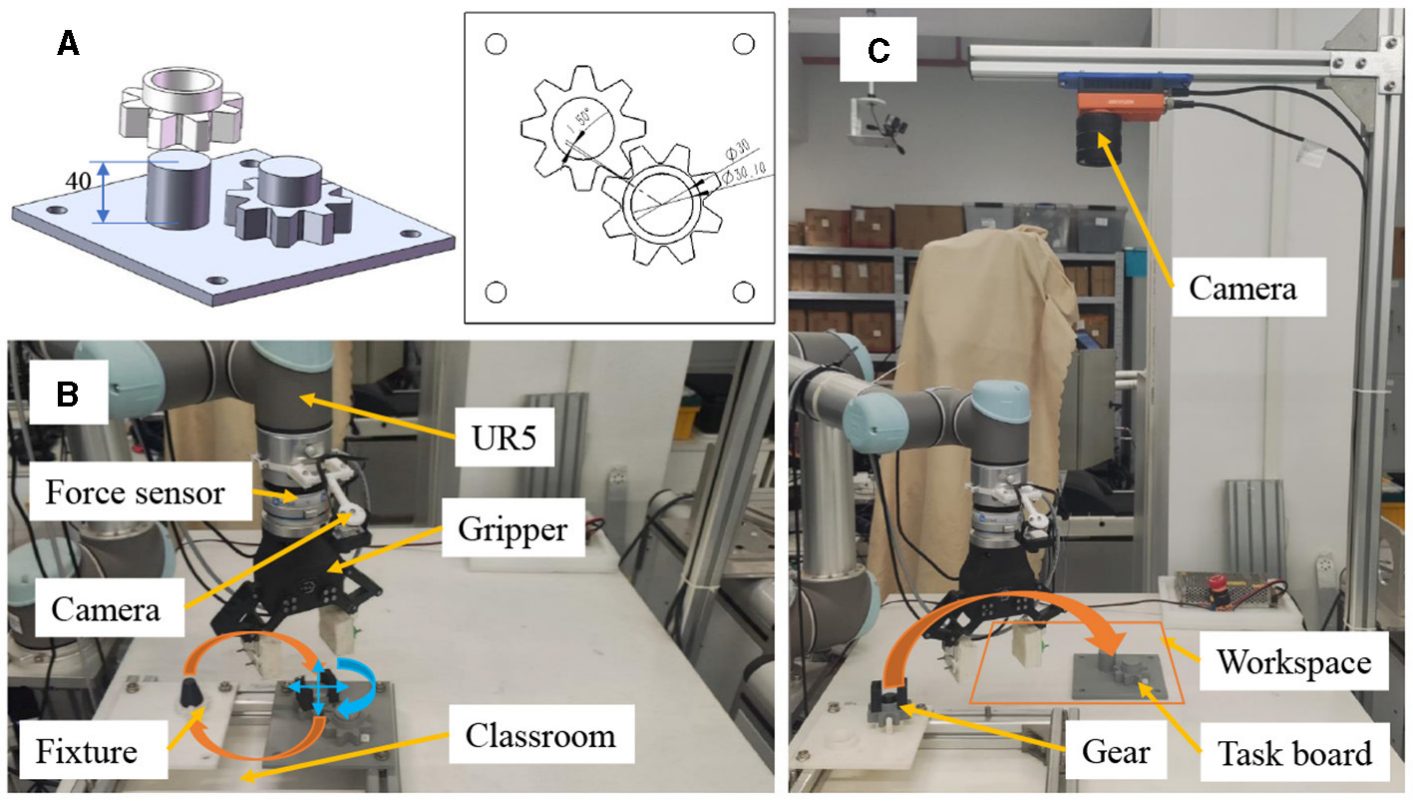
Gear assembly in a semi-structured environment using a UR5 manipulator. **(A)** shows the precise assembly of gears with tight tolerances of less than 0.1 mm and 0.03 radians. **(B)** presents the well-organized environment and hand-designed initialization policy for residual RL. **(C)** illustrates the semi-structured environment used for evaluation.

We investigate the applicability of OEC-IRRL in learning to assemble gears in a semi-structured environment using a UR5 manipulator, as shown in Figure 6. The assembly process involves inserting a gear through a shaft and aligning the wheels with corresponding teeth on another gear. This operation necessitates tight tolerances of less than 0.1 mm and 0.03 radians. The residual policy is initially trained in a structured environment to facilitate easier initialization and subsequently guided by the base policy to replicate the task randomly placed in a workspace. Additionally, before training and executing the assembly task, the slave object is manipulated using a two-finger gripper and a hand-designed policy, being grasped and moved from a fixture to the workspace.

### 5.2 Comparison with existing studies

There is a critical challenge in the field of robotic assembly research—the absence of universally accepted benchmarks and the difficulty in replicating exact task conditions and equipment across different studies. In this study, we have made every effort to compare our control strategies with those from other studies by selecting benchmarks that are as close as possible in terms of task complexity and manipulator characteristics. Where direct comparisons were not feasible, we have provided a detailed discussion of the context in which each control strategy was trained and evaluated. We initially evaluate the performance of our guided RL system by comparing the task setting, sample efficiency, and the results with existing assembly systems to provide a comprehensive assessment of the proposed approach.

Experimental results indicate that our proposed method outperforms existing baseline work broadly, as shown in Table 1. In contrast to the study by Song et al. (2016), our approach leverages one-shot imitation learning to determine the optimal assembly direction and configuration and employs reinforcement learning (RL) to autonomously refine assembly strategies, thereby accommodating a broader range of positional errors and improving the success rate of contact-rich operations without the need for expert-derived experience. Zang et al. (2023) utilize the ProMP method to model global task space strategies from limited demonstration data and subsequently apply Behavior Cloning (BC) to facilitate neural network training for global skill acquisition. Our method advances this approach by extracting geometric information from the demonstration to improve the VMP, enabling global skill learning from a single demonstration. Additionally, our application of RL for fine-tuning strategies in contact-rich tasks results in higher success rates. Jin et al. (2023) introduces a vision-force fusion curriculum learning scheme to effectively integrate features and generate precise insertion policies for pegs with 0.1 mm clearance. Following a similar line of thought, we implement a base policy with an error curriculum to guide RL for direct learning on real robots. Our method extends to handling large pose errors within the workspace through one-shot imitation learning and a general vision model. Although our approach does not achieve the high sample efficiency and success rate in the study by Zhao et al. (2023), our approach minimizes reliance on expert knowledge by similarly segmenting the state space and deriving the base strategy from demonstration data. In addition, the base strategy guides RL to effectively fuse the vision and force for efficient learning contact-rich manipulation instead of visual servo policy. Comparing our method with the baseline from the study by Shi et al. (2021b), our system demonstrates superior performance at variable poses in semi-structured environments by VMP and a general vision model. Despite similar learning costs, our system achieves a higher success rate by incorporating visual information into the residual RL framework. Building on the study by Carvalho et al. (2022); Davchev et al. (2022), we further extract the geometry information to estimate the uncertainty region from the demonstration data, which is used to structure the policy to reduce human demonstrations and interaction with the environment. Overall, our method as a promising automatic assembly method shows great advantages in success rate and human involvement, and the training time is acceptable for many scenarios.

**Table 1:** Comparison with existing studies.

**Baselines**	**Clearance**	**Pose error**	**Coarse policy**	**Fine policy**	**Success rate**
Song et al. (2016)	0.1 mm	8 mm	Hand-designed	Hand-designed	84%
Zang et al. (2023)	0.5 mm	Unfixed	10 demonstrations	-	87%
Jin et al. (2023)	0.1 mm	15 mm	-	100 k	95.2%
Zhao et al. (2023)	-	Unfixed	Hand-designed	5 k	100%
Shi et al. (2021b)	-	2 mm	1 demonstration	200 episodes	91%
Davchev et al. (2022)	0.4 mm	0 mm	1 demonstration	700 episodes	97.9%
Carvalho et al. (2022)	3 mm	Unfixed	5 demonstrations	3 k	60%
Ours	0.1 mm	Unfixed	1 demonstration	300 episodes (15 k)	100%

### 5.3 Bottleneck extraction from demonstration

This study introduces a methodology based on VMMSD for extracting bottleneck poses, which is critical for representing the task structure and enhancing the adaptability of the acquired policy across different positions. This section aims to evaluate whether VMMSD can detect the bottlenecks in demonstrations with different relative poses and tasks with varying geometry. In total, 2 distinct tasks, gear insertion and peg-in-hole, and 20 random relative poses, within a 0.1 m safe area around the task, were employed to evaluate the methodology. The effectiveness is estimated by measuring the distance between the detected bottleneck pose and the goal pose, contrasting it with the ground truth determined by the geometry constraint.

**Figure 7:**
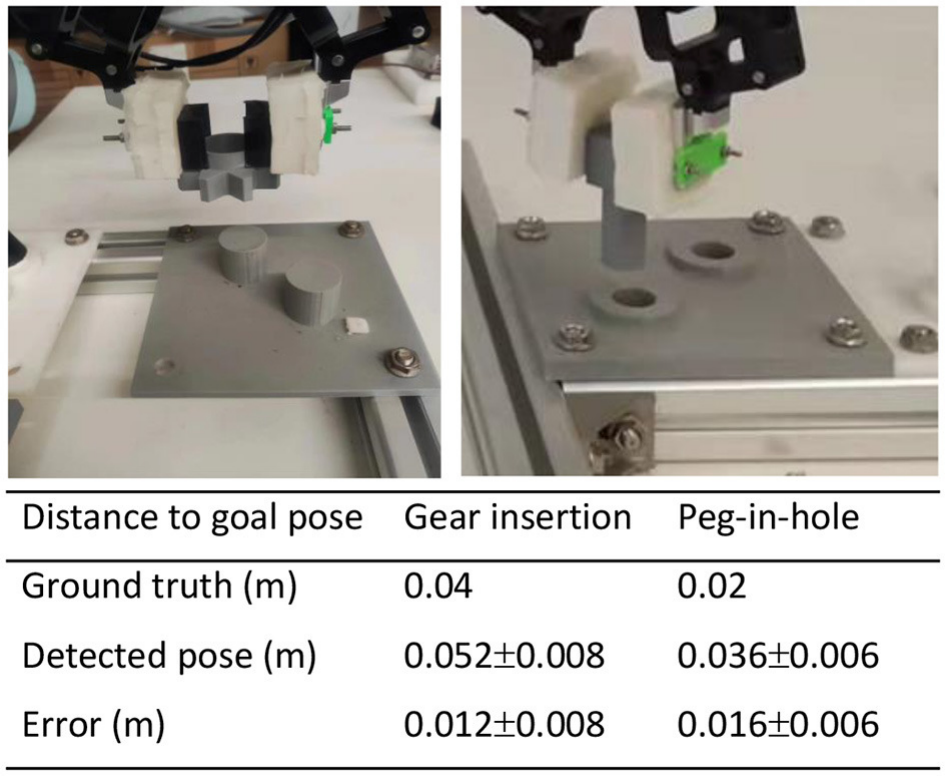
The results of the bottleneck pose extraction for the gear-insertion and peg-in-hole tasks. The distance between the bottleneck pose extracted from the demonstrations and the ground truth is reported as the average value with the corresponding standard deviation.

**Result:** The result shows that the VMMSD can effectively identify bottlenecks in demonstrations, as shown in Figure 7. Compared with the ground truth determined by the geometry (Shi et al., 2021b), the bottleneck poses detected by us show a greater distance, which is the safe area to avoid collisions. It is important to note that the error caused by the safe area will be eliminated by the shape modulation term, and the effect of slightly earlier activation on sample efficiency is deemed acceptable. In addition, the VMMSD method provides a practical alternative that requires a single demonstration and simplifies the identification process compared with learning the variance from multiple demonstrations (Carvalho et al., 2022).

### 5.4 Adaptation of OEC VMP to variable positions

**Figure 8:**
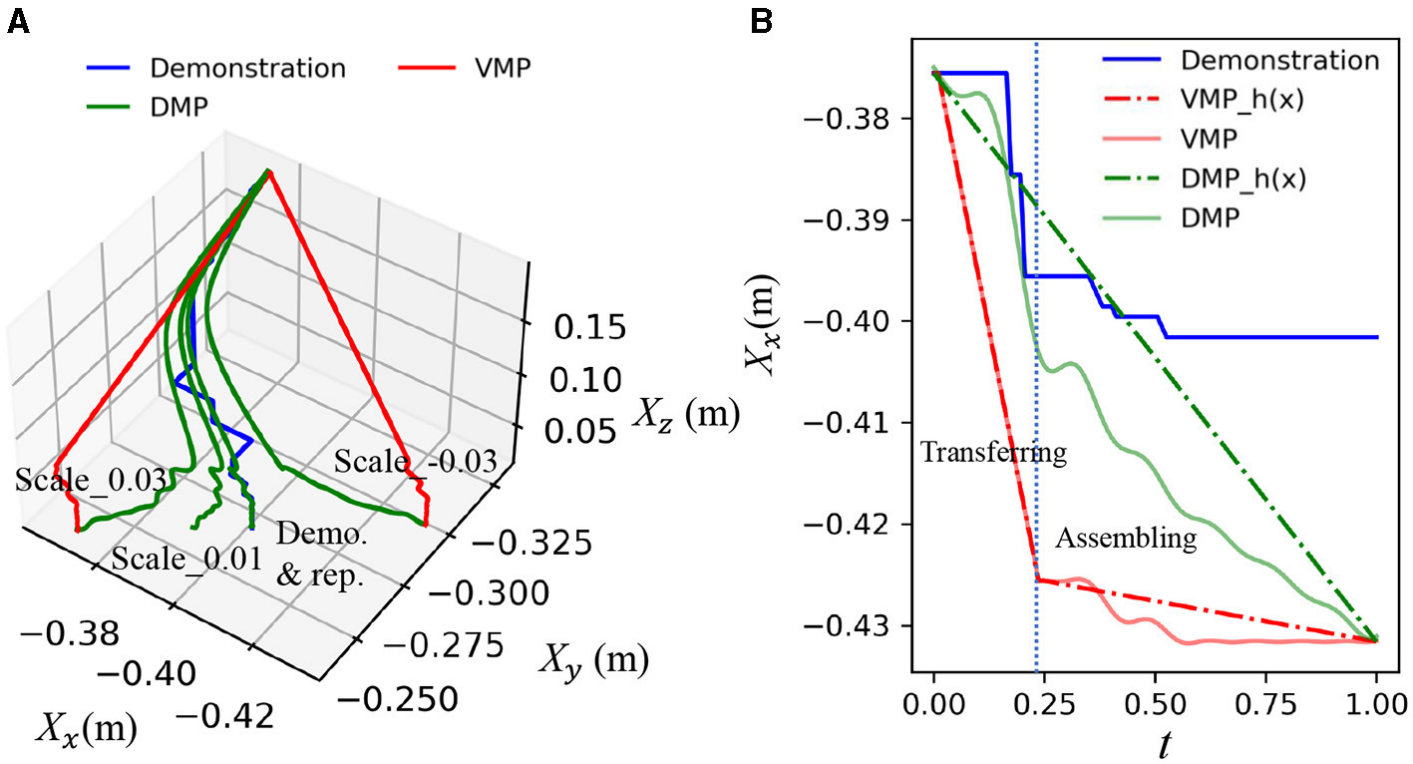
Comparison of VMP and DMP in scaling variable goal poses. **(A)** displays three dimensions of trajectories that were demonstrated and reproduced for translation, illustrating the adaptability of both methods to changes in the goal pose. **(B)** shows the x dimension of trajectories demonstrated and reproduced at a new target point along with the canonical variable *t*, specifically 0.03 meters (m) away from the original teaching target point, to highlight the precision of the scaling.

In this study, we introduce an OEC task representation and VMP to encode the assembly relationship and motion trajectory extracted from a single demonstration and adapt to varying positions in a semi-structured environment. This section aims to evaluate the accuracy of the reproduction and compare it with the DMP (Davchev et al., 2022) without considering the middle via-point. A trajectory, represented by the blue curve as shown in Figure 8, is generated using keyboard teleoperation. As the object's pose changes, the trajectory is regenerated at different positions. We assume that precise guidance during the transferring and assembling phases is essential for efficient residual learning. The correlation distance between the bottleneck pose and the assembly pose is used to measure the loss of geometric information of the regenerated trajectory.

**Result:** The results demonstrate that the OEC task representation and VMP effectively scale the demonstration to varying positions by incorporating the master object pose, as shown in Figure 8. The green curves represent the reproduced and scaled results of the DMP. While the DMP can reproduce the demonstrated trajectory, significant changes in the trajectory profile, particularly when the scaled pose deviates from the demonstrated one, result in the loss of geometric constraint details in the assembling. In contrast, VMP can preserve the integrity of via-points and offer precise motion guidance, as evidenced by the red curve, as shown in Figure 8.

### 5.5 OEC residual RL for contact-rich manipulation

**Figure 9:**
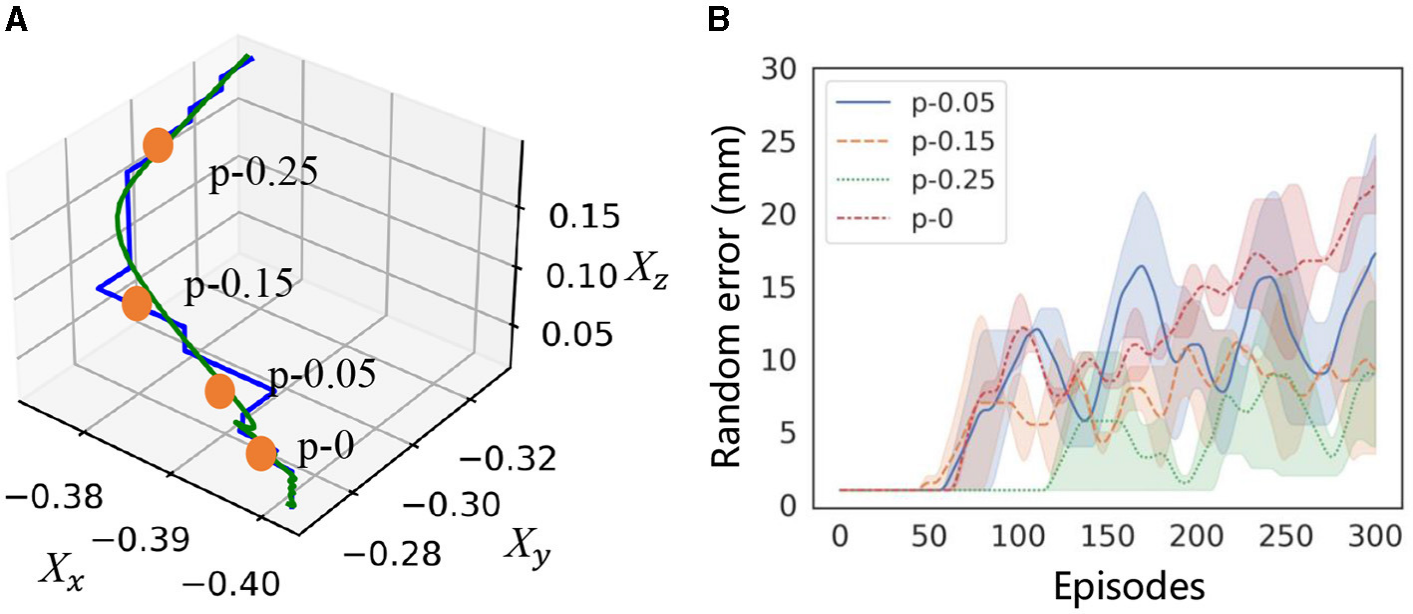
Effect of selective activation on learning efficiency. **(A)** shows the activation poses “p” selected from the demonstration to train the residual policy. **(B)** shows the range of random errors in the curriculum to represent learning efficiency.

Reproducing the trajectory and identifying the activation point to guide the RL agent is crucial for efficient learning and successful application in a contact-rich setting, as it is believed that extensive exploration may cause a decline in performance. This section aims to evaluate how the guidance affects efficiency and whether the OEC-task representation can provide adequate guidance. We compare activation points along the trajectory in learning with an error curriculum, as shown in Figure 9. These points are represented as “p-x,” where “p-0” represents the ground truth of the bottleneck pose determined by geometric information, and “x” represents the distance (m) from “p-0.” The increase of random error in the curriculum is recorded in the training process to measure learning efficiency.

**Result:** The results indicate that the distance between the activation and the ground truth has a significant impact on the learning efficiency, as shown in Figure 9. Comparing error growth, the closest point to the ground truth achieves the best performance. Although the learning efficiency decreases with distance, this decrease is not significant within a range of 50 mm. This suggests that it is feasible to activate residual strategies by extracting the bottleneck pose from the demonstrated trajectory with a distance of approximately 10 mm.

### 5.6 Framework evaluation and comparison with baselines

**Figure 10:**
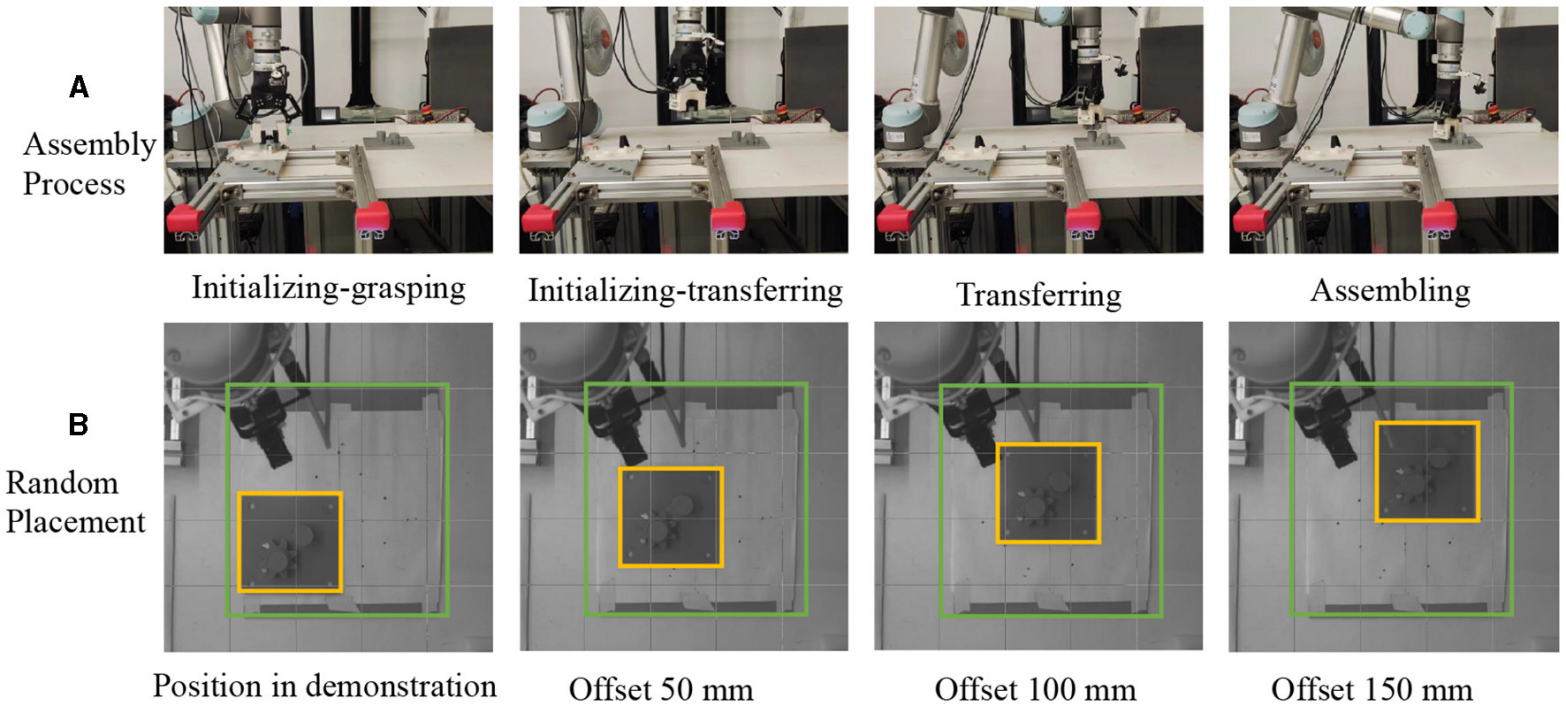
Video stills of the robotic assembly in semi-structured environments. **(A)** shows initialization using a hand-designed policy in a structured environment and manipulation by a learned policy in a semi-structured environment. **(B)** shows the master object placed in the workspace for teaching and testing.

We evaluate the execution of the learned policy in a semi-structured environment by performing a gear assembly task, as shown in Figure 10. The residual policy is trained for 100 episodes, lasts for 1.2 h. We employ a prior YOLO-based pose estimator and OEC task representation to evaluate the impact of VMP as the base policy, comparing it with two other baselines. Notably, we also use only VMP without residual policy as another baseline to illustrate how the hybrid policy can enhance functionally intricate models through synergy. Baseline 1 (Shi et al., 2021a): Visual servo serves as the base policy, with the residual policy consistently active; Baseline 2 (Lee et al., 2020): Model-based trajectory planning is employed as the base policy, taking into account geometric constraints, with the residual policy activated upon reaching the bottleneck pose. Baseline 3: Only VMP is utilized, taking into account temporal and spatial adaptation, but without the residual policy.

In the evaluation, we initialize each episode using a hand-designed policy that contains grasping and transferring to account for the uncertainty introduced by grasping. The base policy for transferring and assembling in semi-structured environments is obtained with only one demonstration. The starting point is outside the workspace so that the image captured by the eye-to-hand camera and the pose estimation by YOLO can avoid the occlusion problem. For robustness evaluation, we introduced variability in several ways to simulate real-world conditions. The master object is placed in the center of a 300 × 300 mm workspace for teaching and anywhere randomly in the workspace for testing. This random placement introduces variability in each episode, requiring the strategies to be adaptable to different conditions. The use of a hand-designed strategy, which includes grasping and transferring actions, introduces variability related to the uncertainty of grasping. This aspect of the experiment simulates the unpredictable nature of real-world object manipulation. We conducted each strategy over 15 executions to statistically evaluate the success rate, the time cost for each episode, and the average contact force. This sample size was chosen to balance the need for a comprehensive evaluation with the practical constraints of experimental time and resources.

**Result:** The results in Table 2 illustrate the effectiveness of the proposed framework in the jigless assembly task. When visual servo is used as the base policy, direct movement toward the goal pose may cause collisions with the target object, thereby lowering the success rate and increasing the contact force in the x and y dimensions. On the other hand, maintaining a constant velocity in model-based trajectory planning results in increased contact force during the search phase, causing larger positional variability and a lower success rate. Compared with baselines 1 and 2, our approach improves the success rate by 46% and reduces the time required by 25%. This improvement is particularly notable as the VMP can learn the geometric constraint and exploratory behavior from a single demonstration. Additionally, the reduced contact force implies a smoother operation and decreased energy consumption. It is important to note that baseline 3, encompassing only coarse operation, was almost unsuccessful in multiple attempts due to uncertainties.

**Table 2:** Comparison of execution with three strategies.

**Task**	**Success rate**	**Cost time (s)**	**Contact force-x (N)**	**Contact force-y (N)**	**Contact force-z (N)**
Baseline 1	0.533	19.000 ± 5.751	1.321 ± 0.453	1.322 ± 0.402	2.978 ± 1.074
Baseline 2	0.733	18.227 ± 4.399	1.101 ± 0.109	1.110 ± 0.146	3.466 ± 1.371
Baseline 3	0.067	23.170 ± 2.345	0.433 ± 0.051	0.120 ± 0.045	3.131 ± 0.705
Ours	1.0	14.920 ± 4.210	1.073 ± 0.102	1.081 ± 0.094	2.208 ± 1.029

Our methodology achieves the best success rates and cost times. The forces during assembly are optimized by reinforcement learning, while baseline 3 has smaller contact forces in the x and y directions due to a lower insertion success rate.

## 6 Discussion

Our experimental results have demonstrated the feasibility of learning a base policy from only one demonstration and a prior vision model to extend residual RL for contact-rich tasks in semi-structured environments. Incorporating additional partial knowledge of the transition function into biomimetic control architectures has a positive effect on sample efficiency, enabling the robot to acquire knowledge akin to that of a well-trained worker based on a specific knowledge architecture. This study introduces an OEC task representation as fundamental common knowledge within the architecture. Imitation learning is demonstrated to be effective in acquiring a base policy from non-expert demonstrations, as evident in two previous studies (Alakuijala et al., 2021; Carvalho et al., 2022). By utilizing the temporal and spatial information provided by fundamental common knowledge, it is possible to learn the base policy of piece-wise VMP from a single demonstration. This approach can adapt to varying positions while maintaining the invariant trajectory for assembly in a semi-structured environment. The use of VMP guidance allows residual RL to account for contact dynamics resulting from unknown physical properties and pose errors due to visual localization and unfixed manipulation, in line with two previous studies (Johannink et al., 2019; Lee et al., 2020). VMP with mode switch detection additionally constrains the exploration space, allowing the agent to perform focused searches around the goal and improving the likelihood of achieving successful exploration toward the goal. The comparison and evaluation results in semi-structured environments suggest that partial knowledge of the transition function is a critical factor for efficient RL in complex tasks, and conversely, RL can facilitate the execution of high-level planning by addressing uncertainty. In other words, the hybrid policy exhibits potential for embodied agents since it enables efficient and safe learning by learning a more powerful known part from low-cost data and the unknown part from interactions. This includes task planning based on large language models (LLMs), which are advanced AI models capable of processing and generating human-like language. LLMs can assist in understanding complex instructions and generating actionable plans for embodied agents, thereby enhancing their ability to perform tasks autonomously (Ahn et al., 2022). While our method has demonstrated enhanced learning effectiveness by leveraging partial knowledge, it is crucial to recognize possible limitations in its implementation. The OEC-VMP-based IL does not account for unforeseen obstacles within the workspace, potentially resulting in dangerous collisions. The proposed approach faces difficulties in generalizing tasks due to the limited number of samples for residual learning.

## 7 Conclusion and future work

This study introduces OEC-IRRL, a framework that improves the sample efficiency of hybrid IL and RL by incorporating additional partial knowledge of transition. The framework proposes an OEC task representation based on a single demonstrated trajectory and a prior vision model, ultimately reducing the number of demonstrations for IL and interactions for RL. OEC-IRRL is designed to be scalable among various task locations. The policy, derived from a single demonstration and less than 1.2 h of interaction, achieves precise assembly tasks in a semi-structured environment with a 100% success rate and an average completion time of 14.92 s. This approach presents a sample-efficient learning-based solution for robotic assembly in flexible manufacturing settings. Future studies will focus on the following areas: (1) Anomaly monitoring and recovery strategies will be explored to ensure the robustness and safety of the system in unstructured environments (Lee et al., 2019); (2) The proposed framework will be utilized to generate more effective real interaction data across diverse tasks for general policy learning through offline reinforcement learning (Hussing et al., 2023) or behavior cloning (Mandlekar et al., 2023).

## Data availability statement

The original contributions presented in the study are included in the article/supplementary material, further inquiries can be directed to the corresponding authors.

## Author contributions

CW: Conceptualization, Data curation, Formal analysis, Investigation, Methodology, Project administration, Software, Validation, Visualization, Writing – original draft, Writing – review & editing. CS: Conceptualization, Writing – review & editing. BS: Conceptualization, Writing – review & editing. GC: Conceptualization, Funding acquisition, Methodology, Project administration, Supervision, Writing – review & editing. LX: Funding acquisition, Supervision, Writing – review & editing.
